# Potential role of irisin in lung diseases and advances in research

**DOI:** 10.3389/fphar.2023.1307651

**Published:** 2023-12-06

**Authors:** Hongna Dong, Xuejiao Lv, Peng Gao, Yuqiu Hao

**Affiliations:** Department of Respiratory Medicine, The Second Hospital of Jilin University, Changchun, Jilin, China

**Keywords:** irisin, lung disease, biomarker, diagnostic, treatment

## Abstract

Irisin, a myokine, is secreted by the movement of skeletal muscles. It plays an important role in metabolic homeostasis, insulin resistance, anti-inflammation, oxidative stress, and bone metabolism. Several studies have reported that irisin-related signaling pathways play a critical role in the treatment of various diseases, including obesity, cardiovascular disease, diabetes, and neurodegenerative disorders. Recently, the potential role of irisin in lung diseases, including chronic obstructive pulmonary disease, acute lung injury, lung cancer, and their associated complications, has received increasing attention. This article aims to explore the role of irisin in lung diseases, primarily focusing on the underlying molecular mechanisms, which may serve as a marker for the diagnosis as well as a potential target for the treatment of lung diseases, thus providing new strategies for their treatment.

## Introduction

Globally, lung diseases, including chronic obstructive pulmonary disease (COPD), bronchial asthma, interstitial pneumonitis, obstructive sleep apnea hypoventilation syndrome (OSAHS), pulmonary embolism (PE), and lung cancer, have high morbidity and mortality rates, adding to the healthcare burden (GBD Chronic Respiratory Disease Collaborators, 2020). Effective measures to enhance the prevention and treatment of lung diseases are urgently required to improve the health of patients and to reduce the healthcare burden.

**FIGURE 1:**
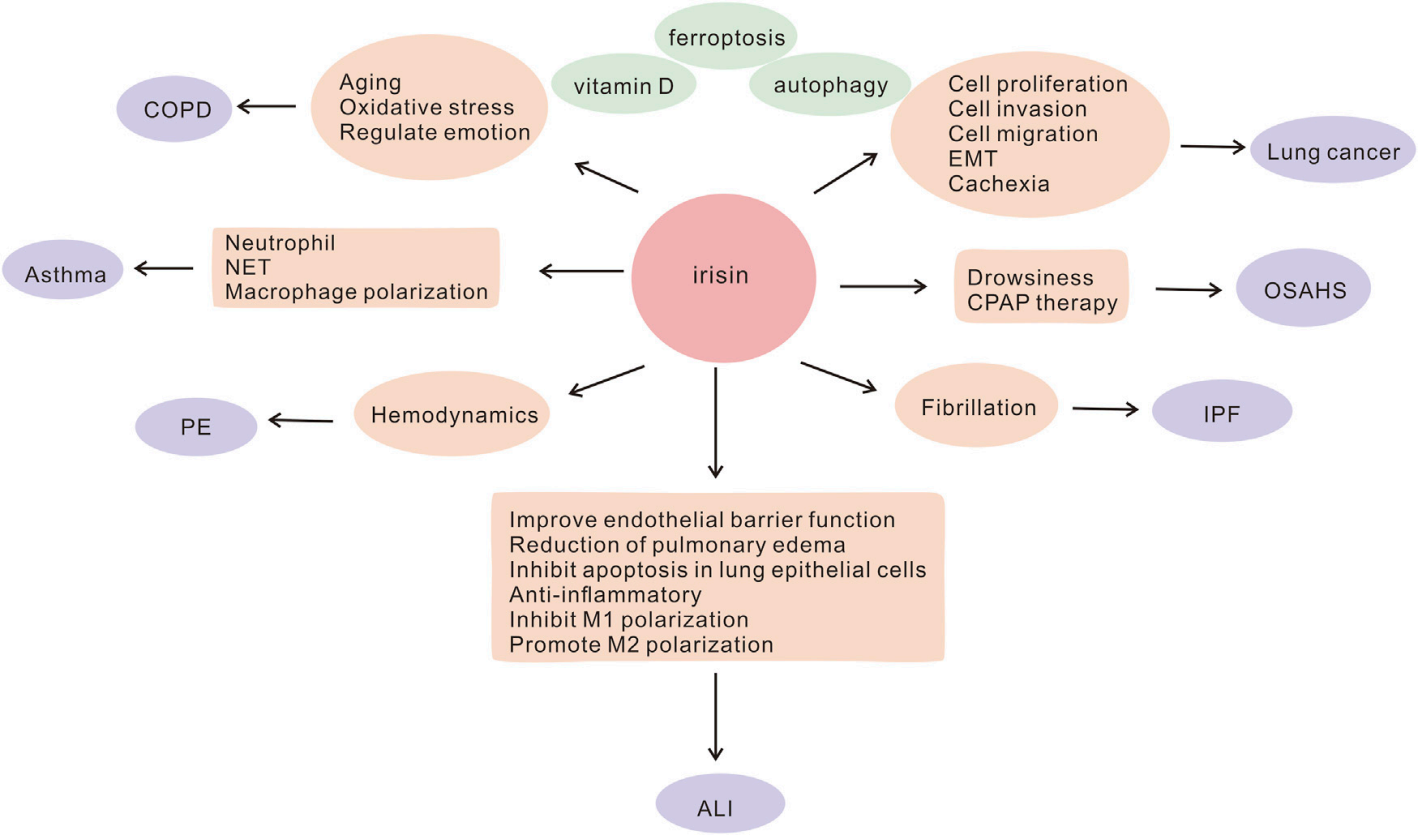
Involvement of irisin in the potential pathogenesis of lung diseases ALI, acute lung injury; COPD, chronic obstructive pulmonary disease; CPAP, continuous airway positive pressure; EMT, epithelial-mesenchymal transition; IPF, idiopathic pulmonary fibrosis; NET, neutrophil extracellular trap; OSAHS, obstructive sleep apnea hypoventilation syndrome; PE, pulmonary embolism.

Irisin, a hormone-like substance, comprises of 112 amino acids. Fibronectin type III domain-containing protein 5 (FNDC5), an irisin precursor, is formed by the cleavage of irisin (Boström et al., 2012). Exercise improves cardiorespiratory fitness, and irisin is primarily secreted by skeletal muscles during exercise. Available literature suggests that soccer players have significantly higher serum irisin levels compared to healthy individuals, which may be attributed to exercise and skeletal muscle function (Gaudio et al., 2021). Irisin is essential for the maintenance of metabolic homeostasis, regulation of energy and heat production, promotion of white fat browning (Wang and Pan, 2016; Xiong et al., 2019; Chen et al., 2021; Aladag et al., 2023), attenuation of insulin resistance (IR) (Ye et al., 2019; Zheng et al., 2022), as well as regulation of bone metabolism (Buccoliero et al., 2021; Zhu et al., 2021) and neurological functions (Pignataro et al., 2021). A growing body of evidence suggests that irisin may have a protective role in lung diseases. Several studies have shown that it is aberrantly expressed in lung diseases and is involved in the pathogenesis of COPD, asthma, acute lung injury (ALI), pulmonary hypertension, lung cancer, and other lung diseases. It acts by reducing oxidative stress (Ho et al., 2018), improving endothelial cell function (Bi et al., 2020), resisting apoptosis, and inhibiting inflammatory factor production (Shao et al., 2017a; Ma et al., 2021). Moreover, irisin inhibits the migration and proliferation of cancer cells and is a potential target for the treatment of lung cancer (Shao et al., 2017b). It may be a potential biomarker and therapeutic target for lung diseases. In this article, we summarize the possible molecular mechanisms and functions of irisin in regulating lung diseases (Figure 1), contributing to a better understanding of the role of irisin in lung diseases, which could help to identify novel targets for the diagnosis or treatment of lung diseases as well as to develop promising interventional strategies for the treatment of these diseases.

## Irisin and cellular pathways

Ferroptosis is an iron-dependent mode of cell death that plays a key role in the pathogenesis of lung disease (Li et al., 2022); therefore, targeting the associated pathways could be a novel strategy for the treatment of lung diseases, including idiopathic pulmonary fibrosis (IPF) (Guo et al., 2023a), ALI (Li et al., 2022), lung cancer (Zhao et al., 2023), COPD (Yoshida et al., 2019), and asthma (Li et al., 2023). Irisin appears to be an important regulator of ferroptosis. Serum irisin expression is reduced in patients with sepsis, with lower irisin levels indicating a higher sepsis severity (Wei et al., 2020). Irisin has an inhibitory effect on ferroptosis through different pathways and beneficial effects on sepsis-related organ damage, such as encephalopathy (Wang et al., 2022) and liver injury (Wei et al., 2020). It has effects on different causes of renal injury. For example, in *in-vivo* and *in-vitro* studies, Zhang et al. have demonstrated that irisin-associated activation of sirtuin 1 (SIRT1)/nuclear factor E2-related factor 2 (Nrf2) inhibits ferroptosis to attenuate renal injury in sepsis patients (Qiongyue et al., 2022). Contrarily, another study has revealed that irisin ameliorates renal injury in ischemia-reperfusion by promoting the ferroptosis protein glutathione peroxidase 4 (Zhang et al., 2021). Meanwhile, in pancreatic cancer, irisin increases ferroptosis and reactive oxygen species (ROS) accumulation as well as helps protect against pancreatic cancer progression (Yang and Leung, 2020). Therefore, irisin may be used to treat lung diseases by modulating ferroptosis-related pathways.

Macroautophagy (or autophagy for short) is the process by which cells remove damaged organelles and their own proteins to maintain cellular homeostasis. It is essential for lung health as well as disease, and it helps with bacterial clearance (Junkins et al., 2013). First, autophagy is an important part of lung development and morphogenesis, and its impairment may lead to bronchopulmonary dysplasia (Yeganeh et al., 2019). Autophagy is not only involved in lung development and maintenance of morphology, but it is also a key link in the development and treatment of lung diseases (Yeganeh et al., 2019; Zhang et al., 2022). It plays a crucial role in ALI (Zhang et al., 2018; Nosaka et al., 2020), asthma (Suzuki et al., 2022), IPF (Gui et al., 2015), lung cancer (Hou et al., 2020), and COPD (Bodas et al., 2019). There is an important link between irisin and autophagy. Irisin enhances the expression of the autophagy protein light chain 3-II in particulate matter less than 2.5 µm in diameter (PM2.5)-induced ALI and decreases the expression of p62 protein, which promotes autophagy and decreases the expression of proinflammatory factors such as tumor necrosis factor-alpha (TNF-α), interleukin (IL)-1β, and IL-18 (Ma et al., 2023). In a diabetic cardiomyopathy model, irisin led to inhibition of autophagy in H9c2 cardiomyocytes and ameliorated IR through the phosphatidylinositol 3-kinase (PI3K)/AKT pathway (Song et al., 2021). In contrast, in C2C12 cells, irisin promoted autophagy and ameliorated IR through the p38–mitogen-activated protein kinase (MAPK)–peroxisome proliferator-activated receptor-gamma coactivator 1-alpha (PGC-1α) signaling pathway (Ye et al., 2019). These contradictory findings are likely due to the use of different cell lines and the complex pathogenesis involved. Irisin also plays a key role in bones and joints. It induces autophagy in bone marrow mesenchymal stem cells, promotes osteoblast differentiation, and plays a role in osteoporosis, as evidenced by the Wnt/β-catenin pathway (Chen et al., 2020). In addition, it has a protective role in degenerative disc disease. Exercise-associated irisin promotes autophagy in nucleus pulposus cells, inhibits cellular senescence and apoptosis, and helps to improve degenerative disc disease (Zhou et al., 2022). By ameliorating cardiac hypertrophy and inducing autophagy through the AMP-activated protein kinase (AMPK)–Unc-51-like autophagy-activating kinase 1 pathway as well as reducing cardiomyocyte apoptosis, irisin may be a novel target for the treatment of cardiac diseases (Li et al., 2018; Li R. et al., 2019). It has a paradoxical role in the regulation of autophagy, possibly due to different microenvironments and acting on different signaling pathways. Therefore, regulation of autophagy by irisin opens up ideas for treating lung diseases and may serve as a new approach to therapy.

Irisin not only plays an important role in regulating ferroptosis and autophagy in lung diseases, but it may be a predictive biomarker for lung diseases. Its aberrant expression in lung diseases is involved in the molecular pathogenesis of lung diseases as well as mood regulation, and it is closely linked to rehabilitative exercises, prognosis, and even treatment. However, further research is required to elucidate the potential role of irisin in lung diseases.

## Irisin and lung diseases

The role of irisin is well established in metabolic, neurological, and cardiovascular diseases (Polyzos et al., 2018; Yu et al., 2019; Qi et al., 2022). Though its role in lung diseases is promising, it has been less studied and requires further exploration. In this article, we summarize the link between irisin and lung diseases as well as its mechanism of action in lung diseases (Table 1).

**TABLE 1:** Expression of irisin in lung diseases and the related molecular mechanisms.

Stimulus	Disease	Tissue/Cell type	FNDC5/Irisin expression level	Signaling pathway	Function and potential role	References
Exercise and smoking	COPD	Serum	Increase (higher than the control and smoking groups)	Nrf2/HO-1	Reduces oxidative stress and improves emphysema	Kubo et al. (2019)
	ARDS	Serum	Decrease	—	Negative correlation with disease severity and mortality	Bi et al. (2020)
LPS	—	Endothelial cells	—	Src-MLCK-β-catenin, AMPK-Cdc42/Rac1	Improves mitochondrial function, enhances barrier function, reduces pulmonary edema, and is anti-inflammatory	Bi et al. (2020)
LPS	ALI	A549 cells	—	AMPK/SIRT1, MAPK/NF-κB	Anti-inflammatory, anti-apoptotic, and improves alveolar epithelial cell dysfunction	Shao et al. (2017a), Li et al. (2019b)
LPS	ALI	MH-S cells	—	HSP90/NLRP3/caspase-1/gasdermin-D	Inhibits M1, promotes M2, attenuates macrophage pyroptosis, and is anti-inflammatory	Han et al. (2023a)
LPS	ALI	A549 cells	—	miR-199a/Rad23b	Anti-inflammatory and reduces lung damage	Ma et al. (2021)
PM2.5	ALI	MH-S cells	—	AMPK/mTOR, Nod2/NF-κB	Enhances autophagy, is anti-inflammatory, and attenuates ALI damage	Jiao et al. (2023), Ma et al. (2023)
	NRDS	Serum/BALF	Decrease/Increase	—	Protects mitochondrial function and is protective against lung injury	Chen et al. (2017)
	Lung cancer	—	—	PI3K/AKT	Reduces lung cancer migration, proliferation, and invasion	Shao et al. (2017b)
	Lung cancer	NSCLC cell lines	Decrease	NF-κB/MDR1	Increases the sensitivity of NSCLC cells to paclitaxel treatment	Fan et al. (2020)
	Asthma	—	—	BDNF	Higher irisin/BDNF levels are associated with improved mood disorders	Szilasi et al. (2017)

## Irisin in ALI/acute respiratory distress syndrome (ARDS)

ALI/ARDS is a severe disease with a high mortality rate. It is caused by a variety of factors, including infection, trauma (Ware and Matthay, 2000), sepsis, and coronavirus disease 2019 infection (Roden et al., 2022; Sang et al., 2022). Its pathogenesis varies depending on the microenvironment of the disease. Recently, the role of irisin in ALI has received great attention. In patients with ARDS, the serum irisin level is less than that of healthy individuals; in addition, as the irisin level increases, the disease is milder and the prognosis is better (Bi et al., 2020). Similarly, the serum irisin levels are reported to be less in patients with neonatal respiratory distress syndrome than in healthy neonates, while its concentrations in bronchoalveolar lavage fluid are less than the serum levels. Moreover, exogenous administration of irisin prior to lung ischemia/reperfusion is reported to be protective (Chen et al., 2017). It is further suggested that irisin plays an anti-inflammatory role in the pathogenesis of ALI. Irisin improves endothelial barrier function and lung permeability as well as attenuates lung edema and inflammation in ALI, possibly through the AMPK/SIRT1 signaling pathway and inhibition of p-Src/myosin light-chain kinase/β-linker protein, activation of AMPK-Ras-related C3 botulinum toxin substrate 1/cell division control protein 42 homolog, and protection of mitochondrial function (Chen et al., 2017; Li et al., 2019; Bi et al., 2020). Similarly, the protective effect of irisin against ALI has been validated by Shao et al. in *in-vivo* and *in-vitro* ALI models. The results demonstrate that irisin suppresses MAPK and induces nuclear factor kappa B (NF-κB) pathways, and simultaneously inhibits apoptosis and inflammation in lung epithelial cells (Shao et al., 2017a). Irisin inhibits the heat shock protein 90/NOD-like receptor family pyrin domain containing 3/caspase-1/gasdermin D pathway, suppresses M1 polarization, promotes M2 polarization, and inhibits macrophage cell pyroptosis (Han et al., 2023). Another study has demonstrated that irisin can exert anti-inflammatory effects in lipopolysaccharide (LPS) and Nrf2-induced ALI by inhibiting miR-199a, thus upregulating downstream upregulated Rad23b (Ma et al., 2021). Irisin also has been shown to ameliorate inflammatory cell infiltration in PM2.5-induced ALI, and this is attributed to promotion of autophagy and inhibition of nucleotide-binding oligomerization domain-containing protein 2/NF-κB (Jiao et al., 2023; Ma et al., 2023). Thus, irisin exerts a protective effect against ALI through a variety of mechanisms and is a potential molecule for the treatment of ALI.

## Irisin in COPD

COPD, a chronic airway obstructive heterogeneous disease, includes chronic bronchitis and emphysema. Its pathogenesis involves oxidative stress, cellular senescence, and inflammatory mechanisms (Barnes, 2021; Barnes, 2022). Its high morbidity and mortality rates have burdened the physical and mental health of patients as well as the healthcare system alike (López-Campos et al., 2016). Infection and smoking are among the most common risk factors for acute exacerbation of COPD. Traditional treatment can effectively control the disease and reduce the risk of acute exacerbation, but exacerbations are still unavoidable due to the heterogeneity of the disease. Multiple factors have been identified as COPD biomarkers, providing a new direction for individualized treatment. Irisin has been recognized as a promising biomarker for COPD. It has been shown that the serum irisin levels are lower in COPD patients than in healthy individuals (Ijiri et al., 2015), and these low levels are involved in the development of emphysema (Sugiyama et al., 2017). The serum levels are reduced in COPD patients who smoke (Kureya et al., 2016), and the levels are reported to be elevated in mice in the exercise and smoking group compared to mice in the smoking-only group. Exercise and irisin ameliorate cigarette-induced emphysema, and this effect is through the Nrf2/heme oxygenase 1 (HO-1) pathway (Kubo et al., 2019). Moreover, COPD mice have reduced skeletal muscle FNDC5/irisin expression, and this is due to the fact that irisin promotes skeletal muscle growth and exposure to cigarette smoke leads to skeletal muscle dysfunction (Zhang et al., 2022).

Aging and oxidative stress are important aspects of COPD pathogenesis, and irisin is closely related to both. Oxidative stress due to oxidative/antioxidative imbalance is a critical link in the pathogenesis of COPD. Many studies have demonstrated that irisin exhibits antioxidant activity that inhibits oxidative stress. For example, exogenous irisin administration has been shown to inhibit oxidative stress as well as ameliorate pancreatic inflammation and fibrosis in chronic pancreatitis (Ren et al., 2020); furthermore, it decreases nicotine-induced oxidative stress and endothelial cell dysfunction (Sarwar et al., 2022). In diabetes-induced cardiac microangiopathy, irisin increases antioxidant protein expression and reduces oxidative stress through activation of the ERK1/2/Nrf2/HO-1 pathway (Zhu et al., 2022). Moreover, exercise-induced secretion of irisin reduces oxidative stress and attenuates cigarette smoke-induced emphysema by activating the Nrf2/HO-1 pathway (Kubo et al., 2019). Nrf2/HO-1 has antioxidant activity and is closely related to irisin, which may attenuate oxidative stress in a variety of other diseases through a spectrum of mechanisms that may have beneficial effects in lung diseases. However, additional studies are required to explore these mechanisms.

Aging reduces skeletal muscle strength, impairs body function, and increases the chance of disease. COPD accelerates aging of the lungs, and exercise can slow this process. Serum irisin levels are significantly lower in the elderly than in young and middle-aged individuals (Chang et al., 2017). Similarly, the serum FNDC5 levels are reported to be reduced in aging mice, and exogenous irisin administration helps to ameliorate aging-associated impaired cardiac function, cardiac remodeling, and inflammation (Hu et al., 2022). Irisin helps to delay osteoblast senescence, and in a study involving patients undergoing total hip or knee arthroplasty, old age was associated with lower serum irisin levels (Colaianni et al., 2021). Moreover, irisin is positively correlated with vertebral and femoral bone density, and serum irisin levels have been found to be lower in osteoporotic patients (Colaianni et al., 2021). Exercise improves muscle mass and function, and resistance training increases blood expression of irisin and enhances muscle strength in the elderly (Kim et al., 2015). In COPD patients, the senescence suppressor gene *klotho* has been found to be expressed significantly less among smokers than nonsmokers, and the levels are positively correlated with irisin; thus, the relationship between senescence and irisin levels in COPD deserves to be investigated (Kureya et al., 2016). Irisin helps to ameliorate aging, and exercise training increases its expression, which may be potentially beneficial for COPD treatment.

## Irisin in asthma

Asthma, a heterogeneous disease, involves chronic inflammation of the airways. Based on the proportion of inflammatory cells in the induced sputum, it is categorized as eosinophilic asthma, neutrophilic asthma, granulocyte-deficient asthma, and mixed-cell asthma. The pathogenesis of different types of asthma varies (Simpson et al., 2006).

### Irisin and macrophages in asthma

Macrophages are important cells in the immune response, and exposure to allergenic stimuli in asthmatics leads to monocyte recruitment to promote an inflammatory response, whereas macrophages in the alveoli act as suppressors to maintain homeostasis (Zasłona et al., 2014). Irisin has a significant effect on macrophage polarization and regulates monocyte infiltration in different microenvironments. In cerebral ischemia, irisin has beneficial effects on neurons by inhibiting monocyte infiltration and microglia activation as well as decreasing the levels of the proinflammatory factors TNF-α and IL-6 (Li et al., 2017). Irisin regulates macrophage function, activity, and polarization. The macrophage activity is regulated by reducing ROS overproduction, thereby exerting anti-inflammatory effects (Mazur-Bialy, 2017). We speculate that irisin may likewise have a protective role in asthma by inhibiting monocyte infiltration in the lungs and promoting the activation of resident alveolar macrophages to exert an anti-inflammatory effect, which is worth investigating in the future.

M1-M2 macrophage polarization affects the asthma inflammatory subtype, with M1 and M2 polarization mainly involved in neutrophilic and eosinophilic asthma, respectively (Robbe et al., 2015). Depending on the microenvironment, irisin has a dual effect on macrophage polarization. In an LPS-induced mouse model of sepsis and *in-vitro* cell- and bone marrow-derived macrophages, irisin induces anti-inflammatory differentiation of M2 macrophages, an effect that has been shown to be mediated by the induction of the peroxisome proliferator-activated receptor gamma-related anti-inflammatory system and the Janus kinase 2-signal transducer and activator of transcription 6-dependent transcriptional activation of Nrf2-associated antioxidant genes (Tu et al., 2023). In addition, irisin-induced M2 polarization enhances osteogenesis in osteoblasts, an effect that may be related to AMPK activation (Ye et al., 2020). Moreover, aerobic exercise can effectively activate the FNDC5/irisin and PI3K/AKT signaling pathways, promote the polarization of M2 macrophages, and inhibit the inflammatory response of the liver after myocardial infarction (Wang et al., 2023). In mice, irisin administration following LPS stimulation leads to inhibition of M1 polarization and promotion of M2 polarization, thus reducing LPS-induced production and secretion of IL-1β, IL-18, and TNF-α, resulting in anti-inflammatory activity and reduced alveolar inflammatory cell infiltration (Han et al., 2023). However, the role of irisin in macrophage polarization in asthma inflammatory subtypes seems to be contradictory, possibly due to different experimental reagents and cells as well as the presence of multiple signaling pathways; the exact reason for this is unclear and requires further investigation.

### Irisin and neutrophils in asthma

Neutrophilic asthma, a hormone-resistant asthma, is insensitive to hormone therapy, and a specific drug for its treatment is still being investigated. Irisin inhibits neutrophil infiltration and IL-1β expression at 24 h after cerebral hemorrhage, inhibits macrophage activation, increases M1 polarization to M2, and is neuroprotective (Wang et al., 2022b). Neutrophil extracellular traps (NETs) are a key component in asthma, and plasma NET biomarkers are reduced in asthmatics compared to healthy individuals and are negatively correlated with lung function (Varricchi et al., 2022). In an *in-vitro* model of neutrophilic inflammation, irisin reduced NET formation, inhibited pancreatitis inflammatory cell infiltration, and attenuated injury via the integrin αVβ5-P38/MAPK pathway (Han et al., 2023). Meanwhile, airway smooth muscle cells express αVβ5 integrin and activate transforming growth factor beta *in vivo*, thus promoting cellular hypertrophy in asthma models (Tatler et al., 2011). Nevertheless, the relationship between irisin and neutrophilic asthma remains to be elucidated.

## Irisin and mental health

Irisin also plays an important role in regulating mood. Owing to physical discomfort, COPD patients often have abnormal moods, including anxiety and depression (Yohannes et al., 2022; Martínez-Gestoso et al., 2022). Irisin has a beneficial effect on improving mood. Serum irisin levels are lower in patients with poor moods, and an association has been reported to be related to brain-derived neurotrophic factor (BDNF) (Papp et al., 2017). Moreover, exercise leads to an increase in serum irisin levels; this effect is observed after 8 weeks of exercise training, and appropriate physical activity may help to improve COPD (Ijiri et al., 2015). The exercise-associated increase in irisin levels leads to an improved quality of life and prognosis in COPD patients (Greulich et al., 2014; Boeselt et al., 2017). Similar to its role in COPD, the irisin-BDNF signaling pathway contributes to attenuation of mood disorders, including anxiety and depression, in asthma patients (Szilasi et al., 2017).

## Irisin in IPF

IPF is a type of interstitial lung disease with an unknown etiology and a poor prognosis (León-Román et al., 2022). There is an inextricable relationship between irisin and fibrosis. It has a protective effect in hepatic fibrosis, renal fibrosis, pancreatic fibrosis, and cardiac fibrosis, and it may be a potential target for therapy (Ren et al., 2020; Pan et al., 2021; Armandi et al., 2022; Yang et al., 2023). In a mouse model of carbon tetrachloride-induced hepatic fibrosis, irisin appears to play a key role and can alleviate endoplasmic reticulum stress and hepatic fibrosis through inhibition of protein kinase RNA-like endoplasmic reticulum kinase-mediated destabilization of heterogeneous nuclear ribonucleoprotein A1 (Liao et al., 2021). In addition, patients with the presence of the FNDC5 rs3480 A>G gene variant have a low prevalence of fibrosis in nonalcoholic fatty liver disease (NAFLD). In hepatic fibrosis, irisin is expressed at higher levels in both the serum and the hepatic tissue, and it has a profibrotic effect that is associated with hepatic stellate cell activation (Petta et al., 2017; Dong et al., 2020). In nonobese, nondiabetic, nonalcoholic patients with fatty liver disease, the role of irisin is reversed. The serum irisin levels are more pronounced in patients with more severe fibrosis, and correlation analyses have demonstrated a positive correlation between irisin and the representative fibrosis markers P type III collagen propeptide and type VI collagen cleavage product, which may participate in the pathogenesis of fibrosis (Armandi et al., 2022). In addition, the administration of nicotinamide ribose to mice with NAFLD has been shown to increase the plasma FNDC5/irisin levels as well as FNDC5 deubiquitination and deacetylation via sirtuin 2 for the treatment of NAFLD (Li et al., 2021). Irisin also reduces renal fibrosis. *In-vivo* and *in-vitro* studies on diabetic nephropathy have revealed that irisin attenuates epithelial-mesenchymal transition (EMT) and fibrosis as well as reduces renal injury by inhibiting the Smad/β-catenin pathway (Yang et al., 2023). Furthermore, irisin attenuates angiotensin II-induced and doxorubicin-induced cardiac fibrosis as well as cardiomyocyte hypertrophy (Chen et al., 2019; Pan et al., 2021). In conclusion, irisin is a promising target for the treatment of fibrotic diseases; however, its effect on fibrosis associated with interstitial lung disease remains to be investigated.

## Irisin in lung cancer

Lung cancer is one of the cancers with a high mortality and morbidity. Cell proliferation, invasion, migration, angiogenesis, EMT, and drug resistance can all contribute to its progression. EMT can promote lung cancer invasion and metastasis (Tang et al., 2019; Wang et al., 2022), and it is associated with drug resistance in lung cancer (Cheng et al., 2020). Irisin expression levels vary in different types of cancers, and its role appears to be contradictory. The serum irisin levels were significantly lower in patients with colon, bladder, liver, and breast cancers than in healthy controls (Provatopoulou et al., 2015; Pazgan-Simon et al., 2020; Taken et al., 2022; Celik et al., 2023), whereas they were elevated in patients with kidney cancer (Altay et al., 2018). There are also differences regarding its effects. On the one hand, it is harmful to humans, with increased levels of irisin in liver cancer tissues without changes in the serum, and promotes cancer development (Shi et al., 2017). On the other hand, irisin is beneficial to humans and inhibits cell proliferation, migration, and invasion in ovarian, glioma, and pancreatic cancer cells (Zhang et al., 2019; Huang et al., 2020; Alizadeh Zarei et al., 2023). In pancreatic cancer, irisin is reported to inhibit EMT via the AMPK-mTOR pathway and to suppress tumor progression (Liu et al., 2018). In addition, irisin is highly expressed in breast cancer tissues and is associated with a longer survival and a good prognosis in patients (Cebulski et al., 2022). Irisin appears to be beneficial in lung cancer by reversing the activity of EMT through the PI3K/AKT pathway, which may be useful in controlling the proliferation, invasion, and migration of lung cancer and controlling cancer progression (Shao et al., 2017b). Importantly, FNDC5 expression is decreased in paclitaxel-resistant non-small-cell lung cancer, and exogenous irisin can increase the sensitivity of lung cancer to paclitaxel, thus contributing to treatment. This effect is mediated through the NF-κB/multidrug resistance protein 1 pathway (Fan et al., 2020). Moreover, cachexia, a muscular dystrophy, is a common manifestation of cancer patients and is associated with a poor prognosis. Irisin appears to be an important influencing factor for cachexia, with high levels contributing to a reduced likelihood of developing sarcopenia in metastasis-free colorectal cancer (Oflazoglu et al., 2022). *In-vivo* experiments demonstrate that irisin ameliorates sarcopenia in aged mice (Guo et al., 2023b). In summary, irisin is crucial in the development of cancer. Therefore, exogenous irisin administration is expected to be a therapeutic agent for targeting lung cancer.

## Irisin in PE/idiopathic pulmonary hypertension

PE is an obstructive disease of the pulmonary arteries, and chronic PE can lead to pulmonary hypertension and right ventricular hypertrophy, a disease with a high morbidity rate. Irisin expression can be used to evaluate the hemodynamic changes in idiopathic pulmonary hypertension, and lower serum irisin levels are associated with higher mean pulmonary arterial pressures and a poorer prognosis (Sun et al., 2021). The serum irisin levels are significantly lower in patients with acute PE than in controls without PE, and they are negatively correlated with the PE severity index. Irisin detection is useful for the diagnosis of PE; however, its sensitivity and specificity are poorer than those of D-dimers (Gurger et al., 2021). Consistently, in a study of patients with acute PE, the group with a higher serum irisin level had a better prognosis, was hemodynamically more stable, and had a lower probability of deterioration (Sun et al., 2020). In summary, irisin can be used as a biomarker for the diagnosis of PE and has some significance in predicting the prognosis of patients.

## Irisin in OSAHS

Irisin appears to play a crucial role in OSAHS, with irisin expression negatively correlating with OSAHS severity (Huang et al., 2020). Excessive daytime sleepiness symptoms are associated with high serum irisin and BDNF levels (More et al., 2019). Continuous positive airway pressure (CPAP) is an important technical tool for the treatment of OSAHS, and studies have shown that receiving short-term CPAP therapy increases irisin levels compared to subtherapeutic CPAP (Ng et al., 2017).

## Irisin and treatment

### Traditional therapeutic drugs

Glucocorticosteroids have anti-inflammatory and immunomodulatory properties. They are one of the main drugs used to treat lung diseases. However, they are associated with various adverse effects, including osteoporosis, muscle atrophy, high blood pressure, and high blood sugar levels, which need to be prevented. It has been reported that irisin has an ameliorative effect on dexamethasone-induced muscle atrophy (Chang and Kong, 2020). Moreover, in hepatocytes, glucocorticoid receptor enhances the transcription of the *FNDC5* gene and the expression of irisin (Kim et al., 2017). Paradoxically, dexamethasone downregulates irisin expression (Mohammed et al., 2019). Meanwhile, *N*-acetylcysteine is an antioxidant that reduces ALI inflammation, asthmatic airway hyperresponsiveness, and emphysema formation as well as attenuates pulmonary fibrosis (Zhang et al., 2014; Breau et al., 2019; Lee et al., 2020; Zhao et al., 2022). It also has an important role in lung diseases and elevates irisin levels as well as attenuates oxidative stress and tissue damage in a diabetes model (Yalçın and Gürel, 2021). Furthermore, long-acting muscarinic receptor antagonists are bronchodilators that are one of the mainstays in the treatment of asthma and COPD. COPD treatment with long-acting muscarinic receptor antagonists can raise the serum irisin levels, and a positive correlation exists between the two, which can lead to an improved prognosis and have a beneficial effect on the patient (Mandal et al., 2018).

### Irisin and vitamin D

Vitamin D is a potential medication for asthma and COPD, and it has been associated with asthmatic lung function, airway hyperresponsiveness, and sensitivity to glucocorticoids, with lower levels suggesting a worse prognosis (Sutherland et al., 2010). Vitamin D supplementation reduces asthma airway hyperresponsiveness and inflammation (Agrawal et al., 2013). Moreover, it decreases ALI and pulmonary emphysema-associated inflammation with beneficial effects (Shi et al., 2016; Hu et al., 2019). In type 2 diabetes, vitamin D supplementation increases SIRT1 and irisin expression as well as improves IR (Safarpour et al., 2020). Interestingly, the serum vitamin D and irisin levels are positively correlated in women with sarcopenia (Wang et al., 2022d). Vitamin D supplementation also increases the serum irisin levels in hyperparathyroidism, as demonstrated in skeletal muscle cells *in-vitro*, probably acting through the SIRT1 and PGC-1α pathways (Sanesi et al., 2023). Thus, vitamin D has a positive effect on irisin, increasing its levels and playing a critical role in various diseases.

### Irisin and other potential therapies

In summary, ferroptosis and autophagy have important contributions in the pathogenesis of lung diseases, and alleviation of lung diseases by drugs that modulate ferroptosis-related pathways and autophagic processes is a potential therapeutic option. The close association between irisin and iron-dependent death as well as autophagy and targeting it opens up the opportunity of treating lung diseases and may serve as a new therapeutic approach worth developing.

## Conclusion

In summary, irisin is closely associated with the diagnosis, treatment, and prognosis of lung diseases, making it an attractive target for the treatment of lung diseases. However, the mechanisms underlying the development and progression of various lung diseases are highly intricate and involve multiple influencing factors and signaling pathways. Exogenous administration of irisin may be a novel strategy for the treatment of lung diseases. Therefore, an in-depth study regarding the role of irisin in the development and progression of lung diseases will help to create new avenues for disease prevention and treatment. Moreover, additional studies are required to elucidate the mechanism of action of irisin in various lung diseases and its potential as a therapeutic target.

## Figures and Tables

**Table:** 

**ALI**	acute lung injury
**AMPK**	AMP-activated protein kinase
**ARDS**	acute respiratory distress syndrome
**BALF**	bronchoalveolar lavage fluid
**BDNF**	brain-derived neurotrophic factor
**Cdc42**	cell division control protein 42 homolog
**COPD**	chronic obstructive pulmonary disease
**CPAP**	continuous airway positive pressure
**EA**	eosinophilic asthma
**EMT**	epithelial-mesenchymal transition
**FNDC5**	fibronectin type III domain-containing protein 5
**HO-1**	heme oxygenase 1
**HSP90**	heat shock protein 90
**IL**	interleukin
**IPF**	idiopathic pulmonary fibrosis
**IR**	insulin resistance
**LPS**	lipopolysaccharide
**MAPK**	mitogen-activated protein kinases
**MDR1**	multidrug resistance protein 1
**MLCK**	myosin light-chain kinase
**NA**	neutrophilic asthma
**NAFLD**	non-alcoholic fatty liver disease
**NETs**	neutrophil extracellular traps
**NF-κB**	nuclear factor kappa B
**NRDS**	neonatal respiratory distress syndrome
**Nrf2**	nuclear factor E2-related factor 2
**NLRP3**	NOD-like receptor family pyrin domain containing 3
**NSCLC**	non-small cell lung cancer
**NOD2**	nucleotide-binding oligomerization domain 2
**OSAHS**	obstructive sleep apnea hypoventilation syndrome
**PE**	pulmonary embolism
**PI3K**	phosphatidylinositol 3-kinase
**PM2.5**	particulate matter less than 2.5 µm in diameter
**ROS**	reactive oxygen species
**Rac1**	Ras-related C3 botulinum toxin substrate 1
**SIRT1**	sirtuin 1
**TGF-β**	transforming growth factor beta
**TNF-α**	tumor necrosis factor alpha
